# Epidemiology of work-related incidents in healthcare: findings from a 7-year retrospective review of work-related incidents in a tertiary hospital in Singapore

**DOI:** 10.1038/s41598-025-23644-1

**Published:** 2025-11-13

**Authors:** Kenneth Bao Ren Leong, Wai Kuen Kam, Shu Rong Lim, Prahlad Govinda Krishnan, Xiaohui Xin, Soo Bee Tan, Alicia S. T. Loi, John Wah Lim, Wee Hoe Gan

**Affiliations:** 1 Department of Occupational and Environmental Medicine, Singapore General Hospital, Singapore, Singapore; 2 Workplace Safety and Health Department, Singapore General Hospital, Singapore, Singapore; 3 Health Services Research Unit, Singapore General Hospital, Singapore, Singapore; 4 Saw Swee Hock School of Public Health, National University of Singapore, Singapore, Singapore; 5 Duke-NUS Medical School, Singapore, Singapore; 6 National Preventive Medicine Residency Programme, National University Health System, Singapore, Singapore; 7 MOH Holdings, Singapore, Singapore

**Keywords:** Occupational safety, Occupational health, Work-related incidents, Healthcare workers, Abuse and harassment, Psychological stressor, Occupational health, Epidemiology

## Abstract

Healthcare workers are exposed to various occupational risks. Due to the high workloads, staff shortages and the COVID-19 pandemic, healthcare workers (HCWs) are at an elevated risk of work-related incidents. This study aims to describe the epidemiology of work-related incidents in a tertiary hospital providing acute care in Singapore; and to analyse those incidents that are of increasing concern. A retrospective descriptive analysis on work-related incidents affecting HCWs in a tertiary hospital in Singapore, between the years 2015 to 2021, was conducted using self-reported incidents from the hospital’s workplace incident reporting system. Across 2015 to 2021, a total of 3306 work-related incidents were reported, regardless of severity of injury. The most common work-related incident was abuse and harassment towards HCWs (23.9%, n = 790). This was followed by slips, trips, falls (23.7%, n = 784) and being hit by / against objects (21.6%, n = 714). The average work-related incident rate nearly doubled from 36.6 per 1,000 staff in 2015 to 70 per 1,000 staff in 2021. The highest increase in incident rate was abuse and harassment, from 4.7 cases per 1,000 staff in 2015 to 22.5 cases per 1,000 staff in 2021. This study reviewed the work-related incident trends in an acute tertiary hospital and highlighted the rising incidence of workplace abuse and harassment towards HCWs. This highlights the current concern of increasing workplace abuse towards HCWs and puts out a call to action to protect healthcare workers and prevent workplace abuse.

**Supplementary Information:**

The online version contains supplementary material available at 10.1038/s41598-025-23644-1.

## Introduction

Every country relies on healthcare workers (HCWs) as an essential resource, yet they face a multitude of occupational hazards^1^. Most recently, HCWs stood at the forefront of the COVID-19 pandemic, supporting the needs of the community while coping with the challenges of an evolving disease, a tight healthcare workforce and shortage of resources from various fronts. Working in the healthcare setting exposed the HCW to various occupational risks, including biological (from exposure to infectious materials), chemical (from exposure to toxic chemicals), ergonomic, physical and psychosocial hazards^2^. Due to high workloads and shortages in staffing, HCWs were at an elevated risk of suffering from work-related incidents that may result in physical injury while having to contend with the stress and emotional demands of working, such as managing challenging interactions with patients and members of the public or experiencing staff burnout^3^.

Prior to the COVID-19 pandemic, there has been an abundance of studies describing the burden and prevalence of workplace injuries in the healthcare sector^4,5^. However, the COVID-19 pandemic has brought about many changes in the healthcare sector which may result in changes in the epidemiology of workplace injuries. Concurrently, a recent scoping review has also revealed that there is a lack of studies investigating or describing the change in epidemiology of workplace injuries or incidents brought about by the COVID-19 pandemic^6^.

Particularly during the COVID-19 pandemic, there had been growing reports of a high prevalence of abuse experienced by HCWs, with workplace abuse being a frequent occurrence ^7,8^. If left unchecked, the rise in abuse and harassment may have wide-reaching impacts on our healthcare systems. Victims of workplace abuse may suffer from post-traumatic symptoms, miss work, or may even think about leaving their jobs or switching careers^9^. Such stressors impact the delivery of care and places additional burden on an already short-staffed workforce.

This study seeks to address the gap in epidemiological data on work-related incidents in a local tertiary hospital occurring during the COVID-19 pandemic and, in particular, the prevalence of abuse and harassment among HCWs. The study aims to identify the trends and patterns in the types of work-related incidents that HCWs face over a 7-year period, from 2015 to 2021.

## Methods

In this retrospective study, we conducted a descriptive analysis on HCWs work-related incidents between 2015 and 2021 at Singapore General Hospital (SGH). SGH is the largest acute tertiary hospital in Singapore, with a capacity of 1,939 beds, 9,201 staff, and oversaw the care of 75,416 patients in 2021^10^.

Data on work-related incidents occurring from the calendar period of 2015 to 2021 were extracted from the incident reporting platform, Risk Management System (RMS) database, in December 2022 by SGH staff from the Workplace Safety and Health Department (WSHD). The extracted data was anonymised and stored in the hospital database for analysis. The RMS is used by the hospital as an operational database to manage unsafe acts and unsafe conditions leading up to work-related incidents, and to learn from the incidents and remedy hazardous conditions to prevent recurrence. A work-related incident was defined as any situation or occurrence at work which resulted in physical or non-physical harm to the staff. Incidents that were reported in the system would be reviewed and verified by assigned individuals, such as the staff’s reporting officers.

As part of the hospital’s policies, all cases of work-related incidents are to be reported on the RMS by the affected staff or their supervisor. The affected staff or supervisor is required to provide incident details such as the date and location of the incident, and a brief description of the incident. Subsequently, the supervisor is required to provide their assessment of the incident and details of the medical leave or hospitalization leave.

Based on the description of the work-related incident reported on the RMS, WSHD categorized each incident into one of seven categories. The seven adopted categories are: (1) abuse and harassment; (2) burns/scald; (3) cut, this excludes sharps injuries caused by needles and tools contaminated by potentially infectious patient body fluids; (4) hit by/against object; (5) occupational health disorders; (6) slips, trips, falls; and (7) spill. Sharp injuries were not available for analysis as they are separately tracked. These categories were adapted from the categories utilised by the Singapore Ministry of Manpower for categorisation of work-related incidents. A broad definition of abuse and harassment, consistent with the legal definition under the Protection from Harassment Act 2014, was adopted for reporting purposes. As such, “Abuse and Harassment” was defined as any threatening or insulting communication or behaviour towards a staff which caused alarm or distress to the staff. A broad definition of Occupational Health Disorders was also adopted for this study and was defined as any health condition that could be attributable to work factors. This includes musculoskeletal conditions, dermatological conditions as well as noise-induced hearing loss. Staff who were reported to be suffering from occupational health disorders would be reviewed by either an occupational health doctor or their own treating physician. Supporting evidence of the diagnosis and assessment would be reported in our incident-reporting database. During data processing, staff designations were categorized into five main occupations: (1) administration, comprising of administrative and management staff; (2) allied health, such as physiotherapists, radiographers, medical laboratory technologists and medical social workers; (3) ancillary, consisting of staff providing support to the overall functioning of the hospital such as kitchen staff, housekeeping staff and laundry staff; (4) medical; and (5) nursing staff.

### Analysis

Descriptive analysis was performed to explore trends in work-related incidents for the period between 2015 and 2021. Incidence rates were calculated based on the total number of work-related incidents occurring in a particular time period per 1,000 healthcare staff working in SGH during the same time period. Data on the total number of healthcare staff by calendar year was obtained from the human resources department, and this was used as the denominator to calculate the person-time at-risk. Confidence intervals were calculated assuming a Poisson distribution.

Ethical approval was obtained for use of deidentified data, as provided by the SingHealth Centralised Institutional Review Board (CIRB 2020-053).

## Results

After exclusion of 134 incidents in the dataset that did not involve staff from the hospital, there were a total of 3,306 work-related incidents reported from 2015 to 2021. As shown in Table 1, the most common work-related incident was abuse and harassment towards HCWs (23.9%, n = 790). This was followed by slips, trips, falls (23.7%, n = 784) and being hit by/against objects (21.6%, n = 714). Among HCWs, nurses accounted for majority of the work-related incidents (50.5%, n = 1,670), followed by ancillary staff (24.4%, n = 808) and allied health staff (17.5%, n = 578).

**Table 1:** Frequency of workplace incidents by occupation and incident category from year 2015 to 2021.

	Occupation					Total n (%)
	Admin n (%)	Allied health n (%)	Ancillary n (%)	Medical n (%)	Nursing n (%)	
Incident category						
Abuse and harassment	24 (14.8)	61 (10.6)	88 (10.9)	31 (35.2)	586 (35.1)	790 (23.9)
Burns/Scald	2 (1.2)	13 (2.2)	23 (2.8)	1 (1.1)	24 (1.4)	63 (1.8)
Cut	8 (4.9)	65 (11.2)	93 (11.5)	8 (9.1)	57 (3.4)	231 (7.0)
Hit by/against object	36 (22.2)	111 (19.2)	226 (28.0)	14 (15.9)	327 (19.6)	714 (21.6)
Occupational health disorders	23 (14.2)	180 (31.1)	86 (10.6)	12 (13.6)	301 (18.0)	602 (18.2)
Slips, trips, falls	68 (42.0)	99 (17.1)	261 (32.3)	18 (20.5)	338 (20.2)	784 (23.7)
Spill	1 (0.6)	49 (8.5)	31 (3.8)	4 (4.5)	37 (2.2)	122 (3.7)
Total n (%)	162 (4.9)	578 (17.5)	808 (24.4)	88 (2.7)	1,670 (50.5)	3,306 (100)

Among nurses, abuse and harassment were the most common work-related incident (35.1%. n = 586), followed by slips, trips and fall (20.2%, n = 338) and hit by / against object (19.6%, n = 327). For ancillary staff, the most common incidents were slips, trips, falls (32.3%, n = 261), followed by hit by / against object (28.0%, n = 226), and cuts (11.5%, n = 93). Occupational health disorders were the most common work-related incident for allied health staff (31.1%, n = 180), followed by hit by / against object (19.2%, n = 111), and slips, trips, falls (17.1%, n = 99).

**Fig. 1:**
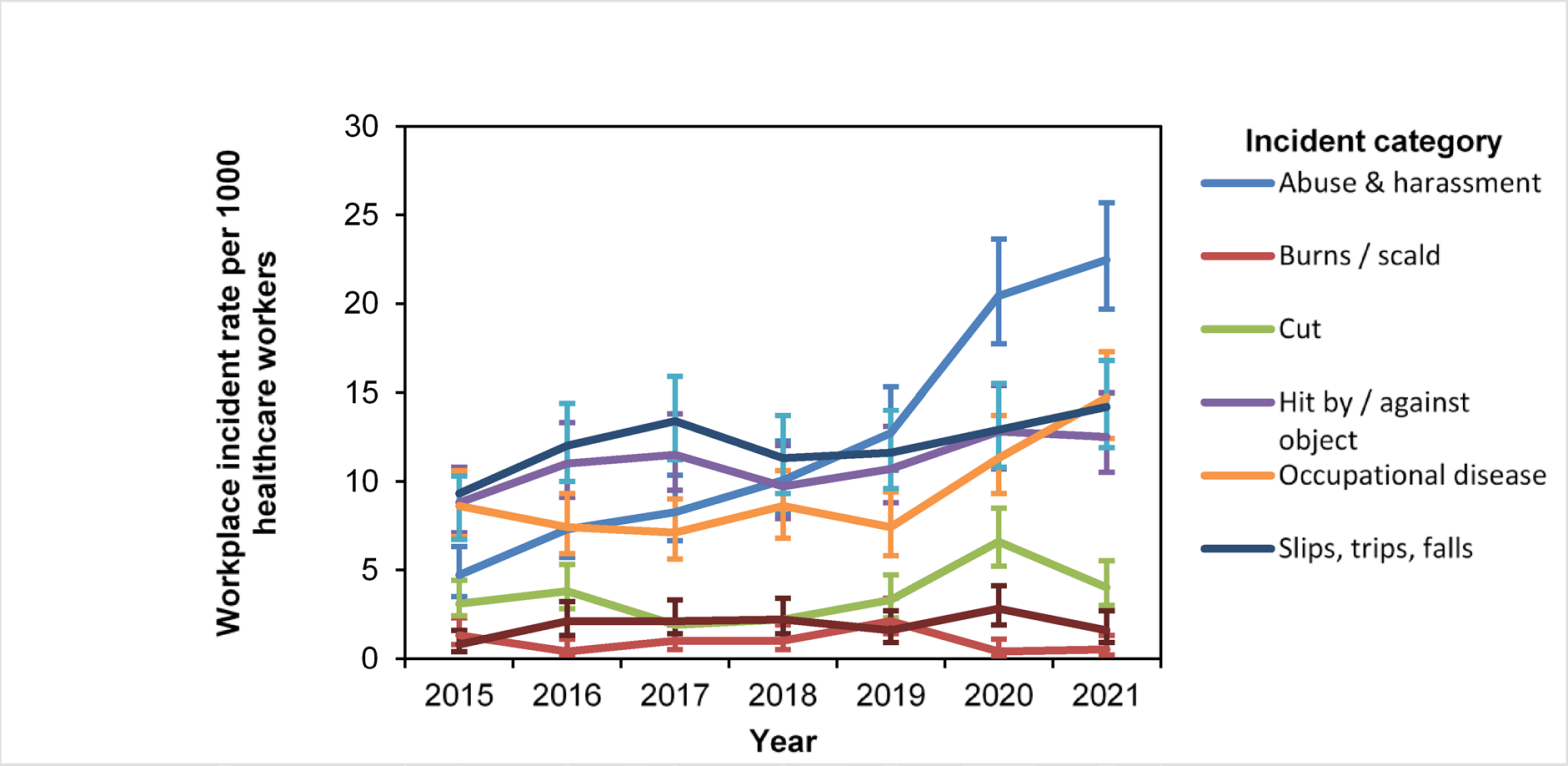
Workplace incident incidence rate per 1,000 healthcare workers by incident category from year 2015 to 2021.

Table 2 shows the incidence rate of work-related incidents nearly doubled from 36.6 per 1,000 staff in 2015 to 70 per 1,000 staff in 2021. The highest increase in incidence rates was for abuse and harassment, increasing from approximately 4.7 per 1,000 HCWs in 2015 to 22.5 per 1,000 HCWs in 2021 (Table 2, Fig. 1). Comparing the incidence rate ratios across types of workplace incidents, there was a statistically significant higher incidence of Abuse and Harassment cases as compared to Burns (0.08 (0.06–0.10)), Cuts (0.29 (0.25–0.34)), Occupational Health Disorders (0.76 (0.68–0.85)) and Spills (0.15 (0.13–0.19) while remaining comparable to Hit by / against object and Slips, Trips, Falls.

**Table 2:** Workplace incident incidence rate (95% CI) per 1,000 healthcare workers by incident category from year 2015 to 2021.

	Year							Incidence rate ratio	
	2015	2016	2017	2018	2019	2020	2021		
Incident category									
Abuse and harassment	4.7 (3.5–6.3)	7.3 (5.7–9.2)	8.2 (6.6–10.3)	10.1 (8.2–12.3)	12.7 (10.6–15.3)	20.4 (17.7–23.6)	22.5 (19.7–25.7)	1.00	
Burns/scald	1.3 (0.8–2.3)	0.4 (1.6–11.1)	1.0 (0.5–1.9)	1.0 (0.5–1.9)	2.1 (1.4–3.4)	0.4 (0.1–1.1)	0.5 (0.2–1.3)	0.08 (0.06–0.10)	*P* = < 0.01
Cut	3.1 (2.4–4.4)	3.9 (2.8–5.3)	1.9 (1.4–3.3)	2.2 (1.4–3.4)	3.3 (2.3–4.7)	6.6 (5.2–8.5)	4.0 (3.0–5.5)	0.29 (0.25–0.34)	*P* = < 0.01
Hit by/against object	8.8 (7.1–10.8)	11.0 (9.1–13.3)	11.5 (9.5–13.8)	9.7 (7.9–12.0)	10.7 (8.8–13.1)	12.8 (10.7–15.4)	12.5 (10.5–15.0)	0.90 (0.82–1.00)	*P* = 0.50
Occupational health disorders	8.6 (6.9–10.6)	7.4 (5.9–9.3)	7.1(5.6–9.0)	8.5 (6.8–10.6)	7.4 (5.8–9.4)	11.3 (9.3–13.7)	14.7 (12.4–17.3)	0.76 (0.68–0.85)	*P* = < 0.01
Slips, trips, falls	9.3 (6.7–10.3)	12.0 (10–14.4)	13.4(11.2–15.9)	11.3 (9.3–13.7)	11.6 (9.6–14.0)	12.9 (10.8–15.5)	14.2 (11.9–16.8)	0.99 (0.90–1.1)	*P* = 0.88
Spill	0.82 (0.41–1.63)	2.1 (1.3–3.2)	2.1 (1.4–3.3)	2.2 (1.4–3.4)	1.6 (0.9–2.7)	2.8 (1.9–4.1)	1.6 (0.9–2.7)	0.15 (0.13–0.19)	*P* = < 0.01
Total	36.6 (33.0–40.5)	44.0 (40.1–48.3)	45.2 (41.2–49.6)	45.0 (40.9–49.5)	49.4 (45.1–54.1)	67.0 (62.2–72.7)	70.0 (65.0–75.4)

As presented in Table 3, from 2015 to 2021, ancillary staff had the highest work-related incident rate of 72.7 per 1,000 HCWs, followed by nursing staff with approximately 60.1 per 1,000 HCWs, and allied health with 51.8 per 1,000 HCWs. Throughout 2015 to 2021, work-related incident rates for nursing and ancillary staff steadily increased, while the work-related incident rates for admin, allied health, and medical HCWs remained relatively stable (Table 3). Comparing the incidence rate ratios across occupational groups, Allied Health (1.7 (1.5–2.1)), Ancillary (2.5 (2.1–2.9)) and Nursing staff (2.0 (1.7–2.4)) had statistically significant higher incidence rates as compared to Admin staff while Medical staff (0.3 (0.3–0.4)) had statistically significant lower incidence rates (Table 3).

**Table 3:** Workplace incident incidence rate (95% CI) per 1,000 healthcare workers by occupation from year 2015 to 2021.

	Year							Average	Incidence rate ratio	
	2015	2016	2017	2018	2019	2020	2021			
Occupation										
Admin	22.9 (14.3–36.7)	30.7 (20.6–46.4)	30.2 (20.2–45.1)	21.2 (13.1–34.5)	25.6 (16.6–39.5)	39.3 (28.0–55.3)	37.7 (26.8–52.9)	29.7 (25.5–34.7)	1.00	
Allied health	37.2 (29.0–47.7)	58.5 (48.1–71.2)	51.3 (41.5–63.6)	56.8 (46.3–69.6)	50.5 (40.7–62.7)	58.1 (47.8–70.7)	50 (41.0–62.1)	51.8 (47.9–56.1)	1.7 (1.5– 2.1)	*P* = < 0.01
Ancillary	52.1 (42.5–64.1)	74 (62.1–87.5)	60.2 (49.7–72.9)	60.1 (49.4–73.1)	66.4 (55.0–80.2)	100.6 (86.7–116.8)	97.7 (84.0–113.6)	72.7 (68.1–77.7)	2.5 (2.1–2.9)	*P* = < 0.01
Medical	10.2 (5.8–17.8)	4.8 (2.2–10.7)	9.0 (5.0–16.2)	8.2 (4.4–15.2)	8.3 (4.5–15.4)	23.1 (16.1–33.1)	7.7 (4.1–14.4)	10.2 (8.3–12.6)	0.3 (0.3–0.4)	*P* = < 0.01
Nursing	41.6 (36.1–48.0)	42.7 (37.0–49.3)	52.5 (46.0–59.9)	53.0 (46.3–60.6)	62.5 (55.2–70.9)	80.5 (72.2–89.8)	97.6 (88.6–107.5)	60.1 (57.9–63.5)	2.0 (1.7–2.4)	*P* = < 0.01

**Fig. 2:**
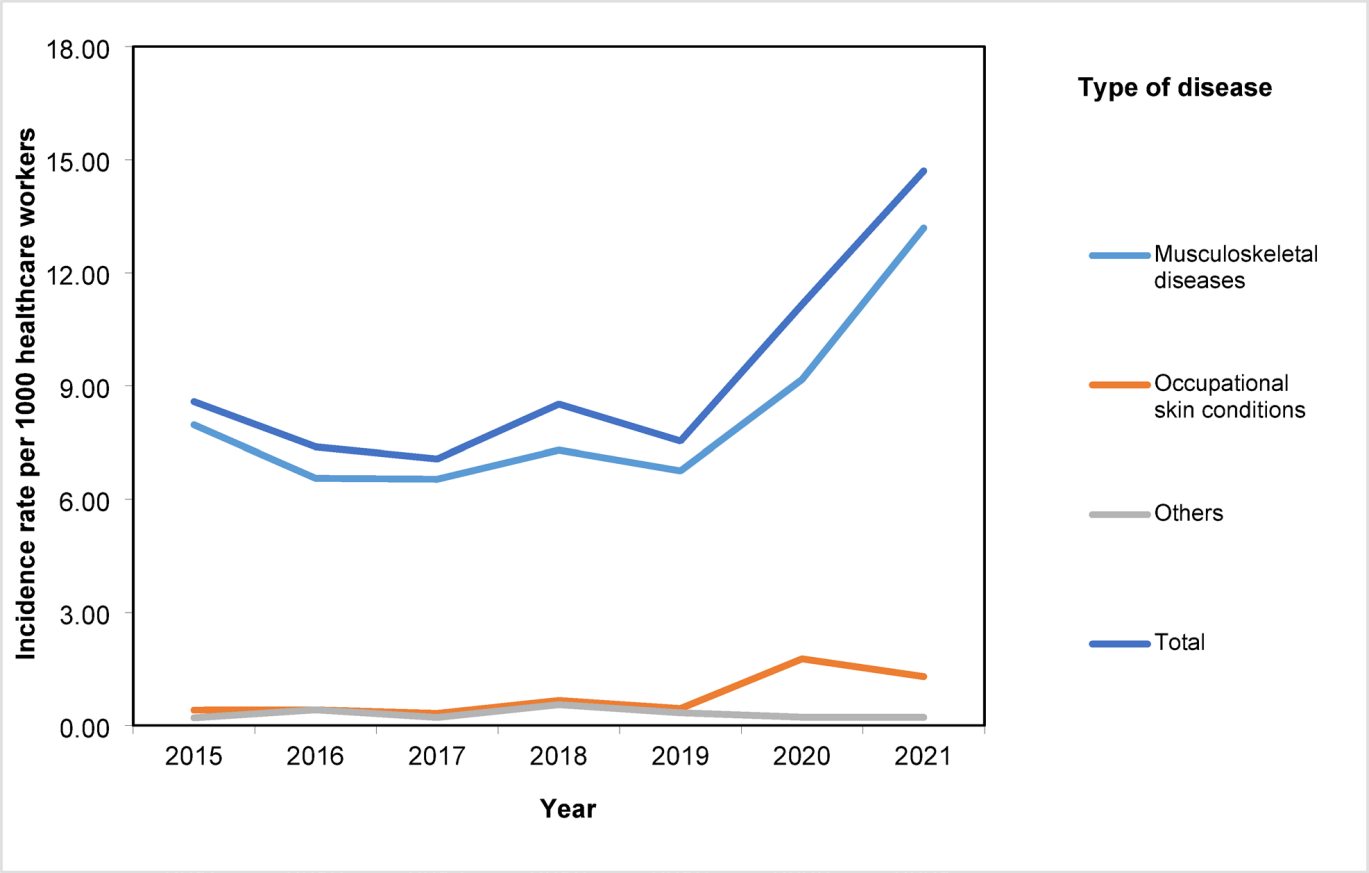
Incidence rate of occupational health disorders per 1,000 healthcare workers by disease type from year 2015 to 2021.

For work-related incidents pertaining to occupational health disorders, as shown in Table 4, musculoskeletal diseases accounted for 88.5% of all incidents (n = 533), followed by occupational skin conditions at 8.1% (n = 49). Figure 2 shows that occupational health disorders incidence rates remained stable from 2015 to 2019 but there was a sharp increase from 2019 to 2021. This was driven by the increased rates of occupational musculoskeletal and skin conditions as shown in Table 5 and Fig. 2.

**Table 4:** Frequency of occupational health disorders by disease type from year 2015 to 2021.

	Year							Total n (%)
	2015 n (%)	2016 n (%)	2017 n (%)	2018 n (%)	2019 n (%)	2020 n (%)	2021 n (%)	
Type of occupational health disorders								
Musculoskeletal diseases	78 (92.9)	63 (88.7)	61 (92.4)	66 (85.7)	60 (89.6)	83 (82.2)	122 (89.7)	533 (88.5)
Occupational skin conditions	4 (4.8)	4 (5.6)	3 (4.5)	6 (7.8)	4 (6.0)	16 (15.8)	12 (8.8)	49 (8.1)
Others	2 (2.4)	4 (5.6)	2 (3.0)	5 (6.5)	3 (4.5)	2 (2.0)	2 (1.5)	20 (3.3)
Total	84	71	66	77	67	101	136	602

**Table 5:** Incidence rate (95% CI) of occupational health disorders per 1,000 healthcare workers by disease type from year 2015 to 2021.

Type of occupational health disorders	Year						
	2015	2016	2017	2018	2019	2020	2021
Musculoskeletal diseases	8.0 (6.4–9.9)	6.6 (5.1–8.4)	6.5 (5.1–8.4)	7.3 (5.7–9.3)	6..8 (5.2–8.7)	9.3 (7.5–11.6)	13.2 (11.1–15.7)
Occupational skin conditions	0.4 (0.2–1.1)	0.4 (0.2–1.1)	0.3 (0.1–1.0)	0.7 (0.3–1.4)	0.5 (0.2–1.2)	1.8 (1.1–2.9)	1.3 (0.7–2.3)
Others	0.2 (0.1–0.8)	0.4 (0.1–1.1)	0.2 (0.1–0.9)	0.6 (0.2–1.3)	0.3 (0.1–1.0)	0.2 (0.1–0.9)	0.2 (0.1–0.9)
Total	8.5 (6.9–9.6)	7.4 (5.9–9.3)	7.1 (5.6–9.0)	8.5 (6.8–10.6)	7.5 (5.9–9.6)	11.3 (9.4–13.8)	14.7 (12.4–17.4)

## Discussion

Our study sought to address the gap in epidemiological data on work-related incidents among HCWs by analysing the trends of workplace incidents captured in our hospital’s incident management database.

Our study showed high incidences of abuse and harassment towards HCWs in a tertiary hospital in Singapore from 2015 to 2021. In addition, the incidence of abuse and harassment had been growing year on year and had become the most common work-related incident. The increasing strain on the healthcare system resulting in longer wait times and greater patient dissatisfaction brought on by the COVID-19 pandemic could have contributed to the increase in abuse and harassment towards HCWs^11^. Additionally, there has been a growing recognition of speaking up and reporting of abuse and harassment towards HCWs^8^. While our study had found the mean abuse and harassment incidence rate of 22.5 cases per 1,000 HCWs in 2021 (Table 2), it is important to contrast this with other sources such as the rates of workplace violence reported by the U.S. Bureau of Labour Statistics (BLS) which found a much lower rate of 14.2 cases per 10,000 HCWs during the same time period^12^. There could be two reasons that account for this disparity. Firstly, the BLS data comprises of cases which resulted in days away from work, job restriction, or transfer away from work. As such, it is likely that only severe cases of workplace violence were captured in the BLS data whereas our hospital-based data is based on a proactive reporting system. Secondly, the definition of workplace violence is narrower than that of abuse and harassment.

Our study revealed that ancillary staff had the highest incidence rates of workplace incidents followed closely by nurses. In the hospital, ancillary staff consists of Patient Service Associates and Healthcare Attendants. Patient Service Associates function as frontline staff attending to administrative needs, such as appointment booking, financial counselling and payments whereas Healthcare Attendents support nurses in the basic care needs for patients. Given that they are a common first point of contact and spend a significant proportion of time attending to patients along with nurses, it is not unexpected for them to be faced with a high risk of workplace incidents such as abuse and harassment. These findings are in line with previous research which also demonstrated that nurses are at high risk for abuse and harassment^13,14^. The nature of the nurse’s work which involves frequent and prolonged contact with patients has been hypothesised as the main reason why they are most prone to abuse and harassment as compared to other HCWs and we believe that the same reasoning could be applied to explain the high rates of workplace incidents seen in ancillary staff. Given the current findings, it is important to address the growing issue of abuse and harassment against HCWs. Victims of abuse and harassment experience decreased job satisfaction and increased levels of stress, decreasing one’s quality of life at work and outside of work^13,14^. In addition, such stressors contribute to poor mental health among HCWs, which has been thought to contribute to suicide among HCWs^15^. With reports of increasing attrition and scarcity of healthcare manpower^16^, this has also become a public health issue and it is of utmost importance that hospitals and governments protect HCWs’ physical and psychological well-being to ensure a well-functioning and effective healthcare system.

Given the rising prevalence of abuse and harassment towards HCWs, many measures have been taken to protect HCWs from abuse. For example, our hospital has enacted a “Code White” protocol^17^ which is activated when a HCW faces a verbal or physical threat from a patient. In response to the rising prevalence, the “Code White” protocol was further enhanced in the emergency department of our hospital in 2022. When activated, a team of four male nurses and two security officers will be alerted to support the healthcare worker under threat. Since its implementation, there have been subjective reports from HCWs that this initiative helped to boost morale and reduced burnout. However, such measures are reactive in nature and more efforts need to be focused on prevention of abuse and harassment towards HCWs and promoting healthy relationships between patients and HCWs. Some examples of this include teaching effective communication skills between HCWs and patients as well as public education campaigns for the public to promote respect for healthcare workers.

Our study also showed a rising incidence of occupational health disorders, such as musculoskeletal diseases and occupational skin conditions, coinciding with the COVID-19 pandemic. Other studies conducted during the COVID-19 pandemic have also found an increased prevalence of musculoskeletal diseases among HCWs^18,19^. This has been hypothesised to be due to the increased workload of HCWs during the pandemic. In the study by Alzeyadi et al.^19^, changes in shift work due to COVID-19 and working in cramped conditions have been found to be significant predictors of musculoskeletal disease. A review of the literature, such as Singapore’s Workplace Safety and Health annual report published by the Ministry of Manpower as well as U.S. Bureau of Labour Statistics data found that overall work-related injuries had decreased during the pandemic in many countries. However, it is important to consider that COVID-19 had caused work restrictions in many industries outside healthcare which could likely have accounted for the overall decrease in work-related injuries reported for the entire working population^20,21^. Similarly, our study found a higher rate of musculoskeletal disorders at 13.2 per 1,000 workers in 2021 while a rate of 11.5 per 10,000 workers was reported in the national workplace safety and health report published by the Ministry^21^. Again, this could be attributed to the possibility that only more severe cases of musculoskeletal disorders were reported to the national registry.

The incidence rates of occupational skin disorders in our hospital ranged from 0.3 to 0.7 per 1,000 person years during the pre-pandemic years. This was similar to the incidence reported in other studies using registry data of occupational skin diseases, which ranges from 0.06 to 0.7 per 1,000 person years^22^. The increase in incidence rates of occupational skin conditions during the COVID-19 pandemic could be attributed to the stricter infection prevention and control measures enforced, such as frequent hand hygiene^23^. This finding serves as a reminder to hospitals that although promoting hand hygiene is important, it is equally important to remind staff to take care of their own health. In this example, this would mean reminding staff to moisturize or apply barrier cream to prevent skin complications from frequent practice of hand hygiene.

Limitations of our study include it being a retrospective descriptive design to investigate work-related incidents extracted from the hospital’s operational database, with the database being affected by staff reporting behaviours. As such, there could be a degree of under-reporting due to behavioural factors such as reporting fatigue or a perceived fear of repercussions. Additionally, our study focused on abuse and harassment arising from patients and did not investigate abuse and harassment among co-workers. Lastly, sharp injuries were not available in the dataset as they are separately tracked in a different database. As sharp injuries are also common workplace injuries in hospitals, it would be important to consider the rates of the workplace incidents studied in relation to the rate of sharps injuries. Despite these limitations, our study adds to existing literature on the incidence and trends of work-related incidents following the COVID-19 pandemic and highlights the need to urgently address the issue of abuse and harassment towards HCWs. The findings of this study can inform the allocation of resources to mitigate the risks faced by HCWs and to improve the work environment of HCWs, which has implications on HCWs’ physical and psychological health.

## Conclusion

Using data from a repository of workplace incidents, this study highlights the pressing need to address workplace abuse towards HCWs, which has now become the most common work-related incident reported in a tertiary hospital providing acute care in Singapore. This complements existing reports of increasing workplace abuse towards healthcare staff^24^ and puts out a call to action to protect HCWs and prevent workplace abuse. It is the right of every HCW to work in a safe and conducive work environment and it is also in such an environment where HCWs can carry out their work productively.

## Supplementary Information

Supplementary Information.

## Data Availability

The dataset used and analysed during the current study is available from the corresponding author on reasonable request.
