# Supplementary material for: Epidemiology of work-related incidents in healthcare: findings from a 7-year retrospective review of work-related incidents in a tertiary hospital in Singapore
Source: Sci Rep. 2025 Nov 13;15:39829. doi: 10.1038/s41598-025-23644-1 (PMC12615818; doi:10.1038/s41598-025-23644-1)
Supplement: Supplementary file 1 — Supplementary Information. [file 41598_2025_23644_MOESM1_ESM.docx]

**Supplementary**

**Supplementary Table 1**. Frequency of workplace incidents by incident category from year 2015 to 2021.

|  | | Year | | | | | | | Total |
| --- | --- | --- | --- | --- | --- | --- | --- | --- | --- |
|  |  | 2015 | 2016 | 2017 | 2018 | 2019 | 2020 | 2021 |  |
| Incident category | Abuse & harassment | 46 | 70 | 77 | 91 | 113 | 185 | 208 | 790 |
|  | Burns / scald | 13 | 4 | 9 | 9 | 19 | 4 | 5 | 63 |
|  | Cut | 30 | 37 | 18 | 20 | 29 | 60 | 37 | 231 |
|  | Hit by / against object | 86 | 106 | 107 | 88 | 95 | 116 | 116 | 714 |
|  | Occupational health disorders | 84 | 71 | 66 | 77 | 66 | 102 | 136 | 602 |
|  | Slips, trips, falls | 91 | 115 | 125 | 102 | 103 | 117 | 131 | 784 |
|  | Spill | 8 | 20 | 20 | 20 | 14 | 25 | 15 | 122 |
| Total | | 358 | 423 | 422 | 407 | 439 | 609 | 648 | 3,306 |
